# Amino acid composition in endothermic vertebrates is biased in the same direction as in thermophilic prokaryotes

**DOI:** 10.1186/1471-2148-10-263

**Published:** 2010-08-31

**Authors:** Guang-Zhong Wang, Martin J Lercher

**Affiliations:** 1 Bioinformatics group, Heinrich-Heine-University, Düsseldorf, Germany

## Abstract

**Background:**

Among bacteria and archaea, amino acid usage is correlated with habitat temperatures. In particular, protein surfaces in species thriving at higher temperatures appear to be enriched in amino acids that stabilize protein structure and depleted in amino acids that decrease thermostability. Does this observation reflect a causal relationship, or could the apparent trend be caused by phylogenetic relatedness among sampled organisms living at different temperatures? And do proteins from endothermic and exothermic vertebrates show similar differences?

**Results:**

We find that the observed correlations between the frequencies of individual amino acids and prokaryotic habitat temperature are strongly influenced by evolutionary relatedness between the species analysed; however, a proteome-wide bias towards increased thermostability remains after controlling for phylogeny. Do eukaryotes show similar effects of thermal adaptation? A small shift of amino acid usage in the expected direction is observed in endothermic ('warm-blooded') mammals and chicken compared to ectothermic ('cold-blooded') vertebrates with lower body temperatures; this shift is not simply explained by nucleotide usage biases.

**Conclusion:**

Protein homologs operating at different temperatures have different amino acid composition, both in prokaryotes and in vertebrates. Thus, during the transition from ectothermic to endothermic life styles, the ancestors of mammals and of birds may have experienced weak genome-wide positive selection to increase the thermostability of their proteins.

## Background

Evolutionary molecular biology is mostly concerned with the forces affecting individual genes. However, observations of variable proportions of guanine and cytosine (GC) in different species and in different genomic regions of vertebrates [reviewed in [1,2]] have prompted the analysis of forces that may affect the evolution of complete genomes. One particular hypothesis concerns adaptation to high temperatures, proposing that high GC content results from selection favouring G:C pairs over less stable A:T pairs [3]. Against initial expectations, there seems to be no direct relationship between the GC content of prokaryotic protein-coding genes and optimal growth temperature [4,5]. Similarly, in the case of vertebrates, it was argued convincingly that the 'isochore' structure of high- and low-GC regions is not due to selection, but reflects varying fixation biases of GC over AT pairs in the presence of recombination [6,7].

A clear picture of selection at work emerges only in the study of structured RNAs. The ribosomal RNAs and transfer RNAs of prokaryotes living at high temperatures contain a much larger GC-fraction in their stem regions compared to homologs from prokaryotes living at more moderate temperatures [4,8], likely because G-C pairs (with three hydrogen bonds) are more stable to thermal fluctuations than A-U pairs (with only two hydrogen bonds). A similar effect is seen in vertebrates: the ribosomal RNA of endothermic ('warm-blooded') animals has a higher GC-content compared to that of ectothermic ('cold-blooded') vertebrates [8,9]. Thus, RNAs that require a specific three-dimensional structure to perform their function appear to be under selection for increased thermostability in cellular environments with elevated temperatures, consistent with the thermal adaptation hypothesis.

However, a higher GC-content in structural RNAs of thermophiles and hyperthermophiles may also have arisen through reasons unrelated to environmental temperatures, e.g., random genetic drift or mutational biases. Closely related species often have similar nucleotide composition and similar habitats simply due to their descent from a common ancestor; a statistically significant relationship between GC content and temperature across species might thus reflect nothing more than a close phylogenetic relationship of these species. This is not the case: even after controlling for phylogenetic relationships, the GC content of structural RNA remains strongly correlated with optimal growth temperature [5]. Thus, genomic effects of thermal adaptation appear to exist at the structural but not the sequence level.

Just like structural RNAs, proteins need to retain their three-dimensional structure in the presence of thermal fluctuations. It hence appears likely that the proteins of thermophilic organisms show corresponding signs of thermal adaptation. Several studies indeed report a correlation between amino acid usage and optimal growth temperature of bacteria [10-14]; however, these studies are based on amino acid usage patterns and not directly on protein thermostability.

Two further analyses are based directly on large datasets of compositional comparisons that took protein structure into account. In a careful study of biophysical properties of a subset of proteins, Glyakina *et al. *[15] confirmed that those amino acids that lead to stronger electrostatic interactions in protein surfaces are enriched among thermophiles, while certain amino acids that tend to de-stabilise proteins are depleted. In another large scale study of the surfaces of hyperthermophilic proteins, Claverie *et al. *[16,17] found solvent accessible charged residues to be strongly overrepresented, concluding that the resulting measure of *CvP*-bias was "the sole criterion that is able to clearly discriminate hyperthermophilic from mesothermophilic microorganisms on a global genomic basis". The measures of amino acid composition derived in the two studies are strongly correlated, as they aim to measure the same phenomenon; they differ only in the treatment of three amino acids.

Thus, amino acid sequence composition is correlated with temperature. However, just as for the GC content of structural RNAs, these correlations could simple be due to the close phylogenetic relationships of some thermophiles and hyperthermophiles. Using the comparative phylogenetic method [18], we show here that patterns of amino acid usage between thermophiles, hyperthermophiles and mesophiles are indeed strongly affected by phylogenetic relationships. Consequently, previous results from direct sequence comparisons are partly misleading. Reassuringly, the two measures of amino acid bias that are derived from studies taking into account the known structure of protein subsets [15,16] are strongly correlated with optimal growth temperature when extended to complete prokaryotic proteomes, even after controlling for phylogenetic non-independence.

Can similar effects of thermal adaptation be seen in higher eukaryotes? The proteins of mammals and birds, which are endothermic species, operate at a species-dependent constant temperature of 35-42° Celsius. This temperature is significantly higher than the average temperature in fish or reptiles, which are ectothermic species. Thus, the same trends observed in prokaryotes may also operate on vertebrate proteins: we hypothesize that compared to ectothermic vertebrates, endothermic animals have proteins with an amino acid composition biased in the same direction as in thermophilic prokaryotes.

Physiological constraints on multi-cellular animals mean that they cannot live at the temperatures in which prokaryotic thermophiles thrive, and thus we expect their amino acid compositions to be less biased. However, the relationship between amino acid composition and thermal stability is approximately linear between 7°C and 103°C [19]. Thus, if thermal adaptation indeed occurred in endothermic animals, it appears likely that the same amino acids as in thermophilic prokaryotes are involved, even if the relevant temperature differences in eukaryotes are substantially smaller than in prokaryotes.

Here, we test this prediction using data from 5 fully sequenced endothermic and 6 fully sequenced ectothermic vertebrates. We first demonstrate that the *ERK *measure [15] of biased amino acid composition shows a strong correlation with optimal growth temperature when applied to genome-scale prokaryotic data, even after controlling for phylogenetic relatedness (as does the *CvP*-bias, see Additional file 1). We then proceed to show that the same measures indicate a weak but statistically significant adaptation of protein thermostability to elevated body temperature also in endothermic vertebrates.

## Results

### Genome-wide bias in amino acid composition of thermophilic prokaryotes

Based on careful structural alignments of 373 proteins, Glyakina *et al. *[15] showed that among the external residues of proteins from thermophilic prokaryotes, three amino acids (E, R and K) are enriched, while seven amino acids (D, N, Q, T, S, H and A) are depleted compared to mesophilic prokaryotes. This effect is quantified by the combined proportion *ERK *= E + R + K - D - N - Q - T - S - H - A (where each letter denotes the fraction of the respective amino acid among all amino acids in a given protein, added for the enriched and subtracted for the depleted amino acids). *ERK *is elevated for the exterior regions of proteins from thermophiles compared to mesophiles [15].

**Figure 1:**
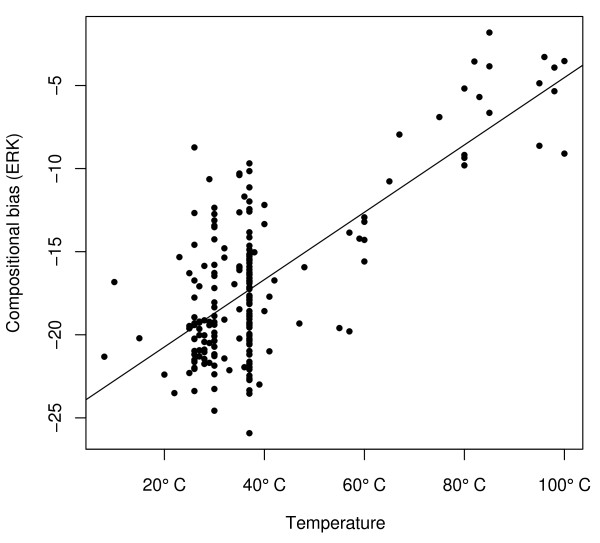
**Amino acid usage is correlated with optimal growth temperature across prokaryotes**. Correlation between a measure of amino acid usage bias that is related to protein stability, *ERK *= E + R + K - D - N - Q - T - S - H - A, and optimal growth temperature in 204 prokaryotes (Pearson's *R *= 0.72, *p *= 10^-15^; Spearman's *ρ *= 0.46, *p *= 10^-12^). See Additional file 1: Supplemental Figure S1 for a correlated alternative measure of amino acid usage bias.

It has not yet been tested if this measure, which was developed from an analysis of external residues, is correlated with the optimal growth (or habitat) temperature of individual species when applied to the full amino acid sequences of complete proteomes [15]. Applying it to complete amino acid sequences dilutes the signal from the surfaces, but is not expected to lead to any systematic biases. To test this, we used a large set of whole genome sequence data and optimal growth temperature (OGT, ranging from 8°C to 100°C) that was previously compiled by Zeldovich *et al. *[20]. This data set contains 204 species (180 bacteria and 24 archaea), of which 16 are hyperthermophiles (optimal growth temperature OGT ≥ 80°C), 11 are thermophiles (OGT = 50-80°C), and 177 are mesophiles (OGT ≤ 50°C). In agreement with earlier observations on the surface regions of a subset of genes [15], we find a strong correlation between the mean *ERK *of complete proteomes and optimal growth temperature (Figure 1, Pearson's *R *= 0.72, *p *< 10^-15^; Spearman's *ρ *= 0.46, *p *= 10^-12^). Analogous results are obtained using *CvP-*bias, a very similar alternative measure of temperature-related amino acid usage (Additional file 1: Supplemental Figure S1, Pearson's *R *= 0.69, *p *< 10^-15^; Spearman's *ρ *= 0.31, *p *= 5.2 × 10^-6^).

As evident from Figure1, this correlation can mostly be attributed to strong differences between hyperthermophiles, thermophiles, and mesophiles (Wilcoxon rank sum tests: *p *= 2 × 10^-5 ^between hyperthermophiles and thermophiles, *p *= 0.00059 between thermophiles and mesophiles, and *p *= 4 × 10^-11 ^between hyperthermophiles and mesophiles; see also Additional file 1: Supplemental Figure S2). However, despite large variation in amino acid composition among mesophiles (Figure 1), we do still see a significant correlation of *ERK *with optimal growth temperature among prokaryotes living at moderate temperatures (between 8°C and 50°C; Pearson's *R *= 0.23, *p *= 0.0017; Spearman's *ρ *= 0.21, *p *= 0.0045). This is in agreement with a detailed study on the properties of six proteins from 42 microorganism living at temperatures ranging from 7°C to 103°C, which found that compositional features related to thermo-adaptation increase almost linearly with temperature [19].

### Amino acid usage patterns are strongly affected by phylogeny

An appreciable number of species in each of the hyperthermophile, thermophile, and mesophile categories are very closely related to each other. The corresponding data points in Figure 1 are thus not statistically independent, and simple correlation statistics as reported above may be misleading. We thus employed the comparative phylogenetic method, which calculates statistically independent contrasts [18]; this eliminates correlations due to common descent. Controlling for phylogenetic relatedness indeed leads to very different patterns of amino acid enrichment/depletion compared to simple correlations (Table 1). In particular, the amino acids A, E, H, I, K, W, and Y, which show significant positive or negative correlations with temperature in a naïve analysis, do not show any significant correlations after controlling for phylogenetic non-independence. In contrast, C, M, N, P, and S, which do not show significant correlations with temperature in the naïve analysis, show significant correlations after including phylogeny into the statistical model (Table 1).

**Table 1:** Pearson's correlation between optimal growth temperature (OGT) and amino acid usage before (*R*) and after (*R*_*Comp*_) controlling for phylogenetic independence.

	Naïve analysis		Comparative method	
**Amino acid**	***R***	***p***	***R*_*Comp*_**	***p***

A	-0.29	0.0021	0.051	0.49
C	-0.056	0.56	-0.27	0.00014
D	-0.27	0.005	-0.40	1.1E-08
E	0.49	4.6E-08	0.092	0.21
F	0.15	0.11	0.0089	0.90
G	-0.086	0.38	0.086	0.24
H	-0.39	3.5E-05	-0.069	0.34
I	0.25	0.0076	-0.084	0.24
K	0.30	0.0013	-0.025	0.72
L	-0.057	0.55	0.096	0.19
M	-0.10	0.29	-0.37	2.1E-07
N	0.033	0.73	-0.29	5.6E-05
P	-0.13	0.19	0.25	0.00049
Q	-0.43	3.3E-06	-0.35	9.5E-07
R	-0.051	0.59	0.27	0.00016
S	-0.10	0.27	-0.40	1.1E-08
T	-0.40	1.4E-05	-0.42	2.6E-09
V	0.17	0.083	0.11	0.14
W	-0.27	0.0030	-0.018	0.80
Y	0.40	1.8E-05	0.081	0.27

In the naïve analysis, there are 6 amino acids (A,D,H,Q,T,W) which are correlated negatively with growth temperature, while 4 amino acids (E,I,K,Y) are correlated positively with growth temperature. After controlling for phylogenetic non-independence, 7 amino acids (C,D,M,N,Q,S,T) are correlated negatively with growth temperature, while only 2 amino acids (R and P) are enriched at high temperatures. Thus, the temperature-related patterns seen for individual amino acids depend strongly on evolutionary history. However, we found that *ERK *and *CvP*- bias, which were both derived including consideration of the protein structure, are still strongly correlated with temperature even after controlling for phylogeny (Spearman's *ρ *= 0.45, *p *= 1.2 × 10^-10 ^and *ρ *= 0.60, *p *= 4.1 × 10^-20^, respectively). These results further underline the importance of structural rather than sequence properties in thermal adaptation.

**Figure 2:**
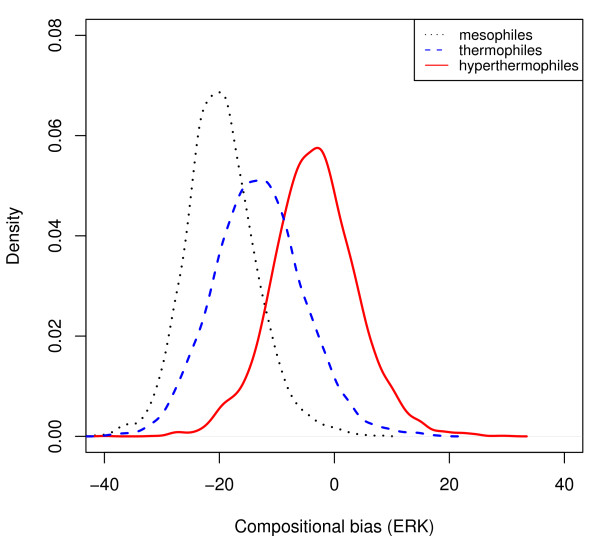
**Amino acid usage differs between mesophilic, thermophilic and hyperthermophilic prokaryotes**. Distribution of amino acid usage bias, *ERK*, across orthologous proteins for 5 species each of hyperthermophilic (optimal growth temperature ≥80° Celsius), thermophilic (50-80°), and mesophilic (≤50°) prokaryotes. All pairwise comparisons are statistically highly significant (Wilcoxon rank sum tests: *p *< 10^-15^). See Additional file 1: Supplemental Figure S3 for a correlated alternative measure of amino acid usage bias. Black: mesophiles; blue: thermophiles; red: hyperthermophiles.

Organisms living at different ambient temperatures may have different protein repertoires, and thus comparisons of complete genomes are potentially misleading. To circumvent this problem, we performed a complementary analysis restricted to groups of orthologous proteins. We collected amino acid sequence data from 5 species each of hyperthermophiles, thermophiles, and mesophiles (Additional file 1: Supplemental Table S1). Using reciprocal best blast hits, we retained only proteins in each group (hyperthermophiles, thermophiles, mesophiles) that had orthologs in at least one of the other groups (see Methods for details). Comparing the remaining 15293 proteins among groups, it is again clear that *ERK*_hyperthermophiles _>*ERK*_thermophiles _>*ERK*_mesophiles _(Figure 2; Wilcoxon rank sum tests: *p *< 10^-15 ^in all pair-wise comparisons). Thus, we confirmed that *ERK*, even when applied to complete amino acid sequences on a genomic scale, is a useful predictor of temperature adaptation in prokaryotes. In the remainder of this paper, we use *ERK *to test for a corresponding effect in vertebrates. We repeated all analyses using the *CvP-bias *[21], in each case obtaining qualitatively very similar results (see Additional file 1); however, as *ERK *and *CvP-bias *differ only in the treatment of three amino acids, these two measures are not statistically independent.

### Endothermic vertebrates have biased amino acid usage

Just as in prokaryotes, the body temperatures of fish, amphibians, and reptiles are closely linked to ambient temperatures. Consequently, the proteins of these ectothermic or 'cold-blooded' vertebrates usually operate below 30° Celsius (Table 2). In contrast, endothermic or 'warm-blooded' vertebrates (mammals and birds) have a thermoregulation system which keeps their body temperatures at a species-specific constant 35-42° Celsius. Does this relatively small difference in temperature between endothermic and ectothermic vertebrates result in a discernible selection pressure for increased thermal stability of proteins? If so, we expect to see compositional biases in the same direction as in prokaryotes, as the rules connecting amino acid usage and thermostability appear to apply across the complete temperature range encountered by life [19].

**Table 2:** Typical temperature ranges for the 11 vertebrate species, and compositional bias of 339 co-orthologs.

Class	Species	T (°C)	ERK	%AT-rich	%GC-rich
**Mammalia**	**Mus musculus**	**36.9**	**-16.32**	**23.22**	**24.56**
**Mammalia**	**Rattus norvegicus**	**37.3**	**-16.27**	**22.82**	**24.91**
**Mammalia**	**Human**	**37**	**-16.11**	**23.82**	**24.31**
**Mammalia**	**Bos taurus**	**38**	**-15.97**	**23.14**	**25.00**
**Birds**	**Gallus gallus**	**39**	**-16.01**	**23.59**	**24.83**
Reptilia	Anolis carolinensis	26 (24- 28)	-17.03	23.75	24.45
Amphibia	Xenopus laevis	21(18-22)	-16.53	25.29	22.52
Amphibia	Xenopus tropicalis	25 (23-28)	-16.53	25.22	22.72
Fish	Danio rerio	28.5	-16.76	23.85	23.45
Fish	Tetraodon nigroviridis	27(25-28)	-16.53	25.22	22.72
Fish	Takifugu rubripes	25(23-26)	-17.17	23.31	23.8

*p*		0.0080	0.0075	0.25	0.13

*p*-values (bottom row) are for comparison of endothermic (mammalia, birds) to ectothermic (reptilia, amphibia, fish) vertebrates, treating each genomic average as a single data point (Wilcoxon rank sum tests). The two last columns list the proportion of AT-rich amino acids (F, Y, M, I, N, K) and GC-rich amino acids (G, A, R, P).

**Figure 3:**
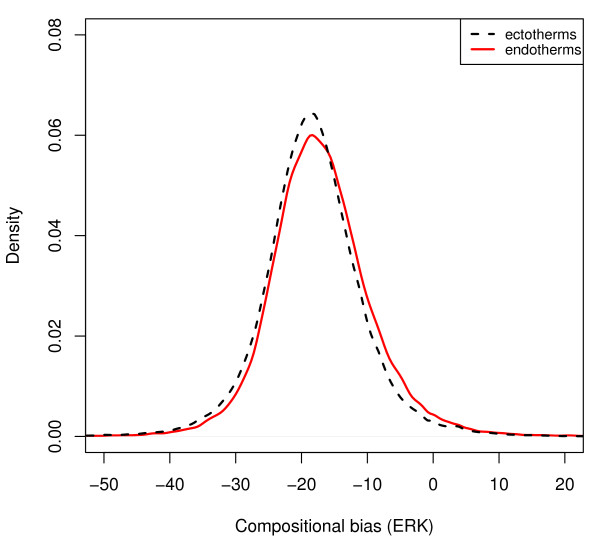
**Amino acid usage differs between endothermic and ectothermic vertebrates**. Distribution of amino acid usage bias, *ERK*, across proteins for endothermic vertebrates (mammals, birds) and ectothermic ('cold-blooded') vertebrates (reptiles, amphibia, fish). *ERK *is significantly increased in endothermic relative to ectothermic animals (Wilcoxon rank sum test: *p *< 10^-15^). See Additional file 1: Supplemental Figure S4 for a correlated alternative measure of amino acid usage bias.

**Figure 4:**
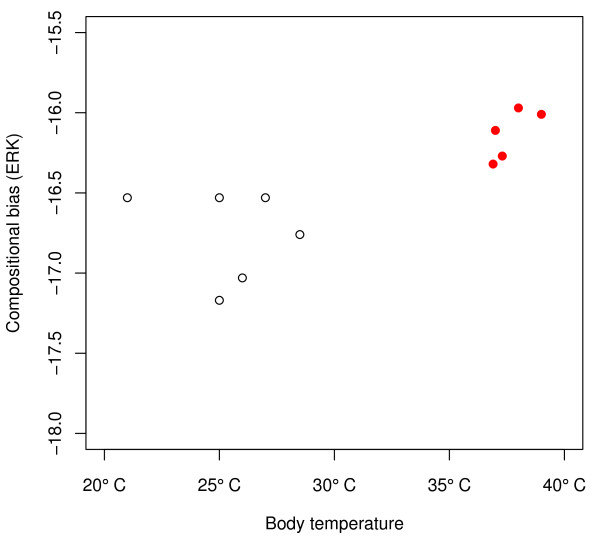
**Amino acid usage bias, *ERK*, in vertebrates is correlated with the typical temperature at which the proteins operate**. Open circles: ectothermic ('cold-blooded') vertebrates; red dots: endothermic ('warm-blooded') vertebrates.

To test this hypothesis of vertebrate thermo-adaptation, we obtained a total of 335,841 protein sequences from 11 sequenced species. This included four mammals: human, rat (*Rattus norvegicus*), mouse (*Mus musculus*), and cow (*Bos taurus*); one bird (the chicken *Gallus gallus*); one reptile (the lizard *Anolis carolinensis*), two amphibia (the frogs *Xenopus laevis *and *Xenopus tropicalis*), and three fish (*Danio rerio, Tetraodon nigroviridis*, and *Takifugu rubripes*). Analysing the combined amino acid composition of the complete proteomes, we indeed find a small but statistically highly significant shift in *ERK *of endothermic compared to ectothermic vertebrates (Figure 3; Wilcoxon rank sum test: *p *< 10^-15^). As shown in Figure 4, there is a strong correlation between the compositional bias of amino acids and the temperature at which the proteins of the species typically act (Pearson's *R *= 0.72, *p *= 0.01). Consistent with our hypothesis, this correlation is mostly due to a systematic difference between ectothermic and endothermic vertebrates (Figure 4).

Again, we confirmed this result by restricting the analysis to orthologous proteins. Among the ectothermic species considered, *Anolis carolinensis *is the closest relative to the endothermic animals and was thus chosen as the reference genome. We identified orthologous proteins in each of the other 10 genomes as reciprocal best blast hits against *Anolis carolinensis*. In pair-wise comparisons (one-sided Wilcoxon rank sum tests, Additional file 1: Supplemental Table S2), all five endothermic species show a significantly higher average *ERK *compared to orthologous proteins in *Anolis carolinensis *(*p *< 0.002 in each comparison), while this is not the case for any of our amphibia or fish (*p *> 0.08 in each comparison).

However, individual proteins in a single species are not truly independent data points, as species-specific compositional biases unrelated to temperature may exist. We thus performed an additional analysis, which treated the average *ERK *across 399 orthologs as a single data point for each of our 11 species. *ERK *is significantly higher for the mammal/bird group compared to the ectothermic group (*p *= 0.0075, Wilcoxon rank sum test on genomic averages, Table 2).

Just as in the prokaryotic analysis, treating closely related species (such as the four mammals) as independent data points could be misleading: similar compositional biases might be due to common descent rather than common physiology. We thus repeated the genome-wide analysis of amino acid bias using the comparative phylogenetic method of independent contrasts. Despite the small sample size, we still find a statistically significant correlation between amino acid bias and temperature after controlling for phylogenetic relatedness (Pearson's *R *= 0.35, *p *= 0.049 for *ERK*, and Pearson's *R *= 0.70, *p *= 0.022 for *CvP*-bias).

### Chicken have elevated *ERK *compared to reptiles

Of all ectothermic animal classes, reptiles - which are paraphyletic due to the exclusion of birds - are the closest living relatives to endothermic vertebrates. Thus, we wanted to confirm that the elevated *ERK *values are indeed restricted to endothermic animals, by comparing the chicken genome to several hundred recently published protein segments of three further reptilia [22]. Based on best blast hits of the segments against the chicken genome, we constructed 508 protein segment alignments between *Alligator mississippiensis *and chicken, 429 segment alignments between *Chrysemys picta *(a turtle) and chicken, and 138 segment alignments between *Anolis smaragdinus *(another lizard) and chicken. *ERK *in chicken protein segments is significantly higher than in each of the three reptilia species (Wilcoxon rank sum tests: *p *= 4 × 10^-16 ^for the alligator, *p *= 0.00027 for the lizard, and *p *= 0.011 for the turtle; see Additional file 1: Supplemental Table S3).

### Elevated *ERK *is not due to biased GC content

The strongest known predictor of amino acid composition at the genomic scale is the GC content of the coding DNA sequences [2,23]. Thus, it is conceivable that the biased amino acid composition (higher *ERK*) in endothermic vertebrates is due to GC content variation between the genomes of endothermic and ectothermic vertebrates. However, for the 339 co-orthologs studied here, there are no differences in the usage of AT-rich or of GC-rich codons between endothermic and ectothermic genomes (Wilcoxon rank sum tests: *p *= 0.25 for AT-rich codons and *p *= 0.13 for GC-rich codons, Table 2).

To further exclude GC content as a confounding factor, we investigated 6227 aligned orthologous coding sequences of human and *Danio rerio *in more detail. As expected, the human genes encoded proteins with significantly higher *ERK *values than their Danio orthologs (Wilcoxon rank sum test: *p *< 10^-15^). If these differences in *ERK *could be fully explained by variation in GC content, we would not expect to see different *ERK *values if we restrict our analysis to those aligned codons that have the same GC content in human and Danio. Contrary to this expectation, we still see higher *ERK *in the human sequences on these GC-neutral codons (Wilcoxon rank sum test: *p *< 10^-15^). Thus, the differences in amino acid composition cannot be simply explained by differences in GC content.

### Elevated *ERK *is not due to purine loading

Secondary structures of RNA sequences are built by the formation of hydrogen bonds between purines (adenine and guanine) and their complementary pyrimidines (uracil and cytosine, respectively). Purine loading, i.e., the over-representation of purines in coding sequences, thus reduces the potential for self-interactions of the mRNA. As self-interactions can interfere with translation, purine loading may be a selected molecular trait. Purine loading is found in almost all prokaryotes, and is positively correlated with optimal growth temperature [reviewed in [2]]. As biased nucleotide composition can lead to biased amino acid composition [2,23], it is conceivable that the observed elevated *ERK *levels in endothermic vertebrates may be a consequence of purine loading.

To exclude purine loading as a confounding factor, we employed an analogous strategy as for GC content. When we restrict the alignments of the 6227 human - *Danio rerio *orthologs to those codons with the same purine content, we still observe a significantly higher *ERK *value in the human sequences (Wilcoxon rank sum test: *p *< 10^-15^). Thus, the biased amino acid composition of proteins from endothermic vertebrates cannot be attributed to purine loading alone.

## Discussion

Building upon earlier results on aligned structures of prokaryotic protein pairs [15,16], we show that genome-wide amino acid usage biases (measured by *ERK *or *CvP*) correlates strongly with the optimal growth temperature of bacteria. That *ERK *and *CvP *measures are derived directly from physicochemical considerations [15,16] strengthens the notion that it is indeed selection on thermostability which is responsible for this long-recognised trend. While the enrichment or depletion of individual amino acids in (hyper-)thermophilic species is strongly affected by phylogenetic non-independence, the overall biases measured by *ERK *and *CvP *are robust.

Applying the same methodology to 11 vertebrate species, we find that mammalian and bird proteomes show a weak but significant increase in *ERK *and *CvP*-bias compared to ectothermic fish, amphibia and reptilia. This increase cannot simply be explained by biases in nucleotide composition, and remains statistically significant when controlling for phylogenetic non-independence. While the examined dataset of genome sequences is necessarily small and not evenly sampled across vertebrates, we thus have strong evidence for a direct relationship between amino acid bias and the temperature at which vertebrate proteins operate. Analogous to the situation in prokaryotes, our findings are most parsimoniously explained by selection for increased stability against thermal fluctuations in endothermic vertebrates. Why then do we not see a correlation of amino acid usage bias with environmental temperature when considering only ectothermic vertebrates (open circles in Figure 4, Pearson's *R *= -0.20, *p *= 0.77)? Apart from an issue of small sample size, this lack of a correlation may be due to the fact that ectothermic vertebrates can rapidly switch between habitats of different temperatures during evolution. This is evident, *e.g*., from the two Xenopus species in our study, which thrive at 18-22 and 23-28°C, respectively (Table 2).

It should be pointed out that ectotherms are not necessarily cold-blooded, i.e., body temperature in some ectothermic species can reach temperatures as high or higher as in endotherms. Furthermore, internal temperature can vary between different body regions of an ectotherm, and can be above the outside temperature [24]. However, the temperatures listed in Table 2 are 'optimal' temperatures for these species, and internal temperatures will indeed be close to these values. On average, body temperature in endotherms is higher than in ectotherms, and has likely remained stable since the last common ancestors of mammals and of birds.

A shift towards stability-increasing amino acids in proteins of endothermic vertebrates mirrors similar effects seen for the nucleotide composition of structural RNAs [9]. While the effect for structural RNAs appears to be much stronger, this may not be surprising: RNA structures are formed by direct bonds between complementary bases, G-C bonds being more stable than A-T bonds. Thus, thermostability of RNAs is directly related to the GC fraction of sites involved in bond formation. The effect of individual amino acids on the thermostability of proteins is much more subtle: the relevance of different physicochemical properties of amino acids depends on their three-dimensional context within the protein structure. The subtleness of this effect was already seen in prokaryotic proteins (Figure 1), where we found only a weak (though significant) correlation of amino acid usage bias with optimal growth temperature among mesophiles.

## Conclusions

Taken together, our results indicate weak but significant genome-wide positive selection on protein structure during the change from ectothermic to endothermic life styles in vertebrates. This molecular process may have been very similar to the adaptation of microorganisms that switch from mesophilic to thermophilic life styles [25], except that the temperature differences involved were much smaller.

## Methods

### Data sources

The genomes of prokaryotic species were obtained from NCBI ftp://ftp.ncbi.nih.gov/genomes/Bacteria. Optimal growth temperatures were taken from Mizuguchi *et al. *[26] and Suhre and Claverie [17], except for *Chloroflexus aurantiacus *J-10-fl, which was obtained from http://genome.jgi-psf.org/finished_microbes/chlau/chlau.home.html.

Genome sequences for *Bos taurus, Danio rerio, Gallus gallus, Homo sapiens, Mus musculus, Rattus norvegicus, Xenopus laevis, Xenopus tropicalis, Danio rerio Tetraodon nigroviridis*, and *Takifugu rubripes *were obtained from NCBI ftp://ftp.ncbi.nih.gov/genomes/ and ENSEMBL http://www.ensembl.org/info/data/ftp/. Protein sequences of Anolis carolinensis were downloaded from the superfamily database http://supfam.mrc-lmb.cam.ac.uk/SUPERFAMILY/index.html. Three sets of non-avian reptile protein coding sequences were taken from Shedlock et al. [22].

### Calculation of amino acid usage bias in prokaryotes

For each protein in each of the 204 prokaryotic species, we calculated *ERK *= E + R + K - D - N - Q - T - S - H - A; here each capital letter on the right hand side stands for the proportion of this amino acid relative to all amino acids in the protein sequence [15]. Total *ERK *for each species was obtained analogously from the concatenated sequences of all proteins. As an alternative measure of amino acid usage bias, we similarly calculated *CvP-bias *= D + E + R + K - N - Q - T - S [21].

To control for biases in gene repertoires of the different life styles, we first chose 5 representative species from each life style group (hyperthermophiles with optimal growth temperature OGT ≥ 80°C, thermophiles with OGT = 50-80°C, and mesophiles with OGT ≤ 50°C). For these 15 species, we performed an all-against-all protein blast search, identifying pair-wise orthologs through reciprocal best blast hits. We then excluded all proteins that had no orthologs outside their group (hyperthermophiles, thermophiles, mesophiles). All remaining proteins had at least one ortholog outside their life style group, and where hence retained for our comparison (Additional file 1: Supplemental Table S1). We then compared mean *ERK *values between groups using Wilcoxon rank sum tests.

### Calculation of amino acid usage bias in 11 vertebrates

*ERK *and *CvP-bias *were calculated as for prokaryotes. As reptiles are the closest relatives of endothermic animals in our set of ectothermic species, we chose the lizard *Anolis carolinensis *as a reference point. We first identified orthologous protein pairs between Anolis and each of the other 10 vertebrates through a search for reciprocal best blast hits. If an *Anolis carolinensis *protein had orthologs in each of the other ten genomes, we included this protein in our co-ortholog list, resulting in 339 groups of ubiquitous orthologs.

### Application of the comparative phylogenetic method

To control for phylogenetic non-independence, we calculated independent contrast using the AOT module implemented in the software Phylocom [18]. In total, 109 species with OGT ranging from 15-100°C were included (Additional file 1: Supplemental Table S4). The phylogenetic tree for these 109 species was obtained from the Tree of Life project [27]. Branches of length 0 were set to 0.00003, which is half the length of the shortest non-zero branches.

To apply the comparative phylogenetic method to the vertebrate data, we first reconstructed the phylogenetic tree. Multiple alignments of the 399 co-orthologs across the 11 species were obtained using Muscle [28]. We eliminated poorly aligned positions with Gblocks [29]. A phylogenetic tree of these 11 vertebrates was constructed from the contatenated amino acid sequences with Phyml [30] using standard settings.

We employed a randomization test to assess statistical significance of the correlation between the independent contrasts. We first calculated Pearson's correlation coefficient *R *for the contrasts of temperature (Table 2) and *ERK*. We then randomised the association between the two types of contrasts and re-calculated the correlation coefficient *R*_*rand*_. This was repeated 9999 times; in 498 randomised data sets *R_rand_*. was equal to or larger than *R*. Treating the observed data as an additional data point, the *p*-value of the correlation was then estimated as 499/10000.

### Comparison of Chicken with three non-avian reptiles

Based on well-aligning best blast hits of protein segments against the chicken genome (protein blast e-value < 10^-5^), we constructed 508 protein segment alignments between *Alligator mississippiensis *and chicken, 429 segment alignments between *Chrysemys picta *(a turtle) and chicken, and 138 segment alignments between *Anolis smaragdinus *(another lizard) and chicken. As before, *ERK *and *CvP-bias *were calculated for each aligned segment.

### GC content and purine content as confounding factors

To check if nucleotide content variation could explain the higher *ERK *values in endothermic compared to ectothermic vertebrates, we performed a detailed analysis of orthologs between human and *Danio rerio*. Orthologs were identified from reciprocal best blast hits. We aligned the translated orthologous protein sequences using MUSCLE [28] with default settings, and then replaced the aligned amino acids with their encoding codons to obtain DNA alignments.

To exclude the influence of differences in GC content between human and Danio on amino acid usage, we then re-calculated *ERK *from only those aligned codons that had the same G+C content in both species. Similarly, to exclude the influence of differences in purine content, we re-calculated *ERK *from only those codons that had the same A+G content in both species.

## Authors' contributions

GW conceived of the study and performed the analyses. GW and MJL designed the study, interpreted the results, and drafted the manuscript. Both authors have read and approved the final manuscript.

## Supplementary Material

Additional file 1 **Supplemental Figures and Tables**. Additional results that support the conclusions of the main text, presented in the form of figures and tables. Click here for file

## References

[B1] Eyre-Walker A Hurst LD The evolution of isochores Nature Rev Genet 2001 2 7 549 555 10.1038/35080577 11433361

[B2] Hickey DA Singer GAC Genomic and proteomic adaptations to growth at high temperature Genome Biol 2004 5 10 117 10.1186/gb-2004-5-10-117 15461805 PMC545586

[B3] Wada A Suyama A Local stability of DNA and RNA secondary structure and its relation to biological functions Prog Biophys Mol Biol 1986 47 2 113 157 10.1016/0079-6107(86)90012-X 2424044

[B4] Galtier N Lobry JR Relationships between genomic G+C content, RNA secondary structures, and optimal growth temperature in prokaryotes J Mol Evol 1997 44 6 632 636 10.1007/PL00006186 9169555

[B5] Hurst LD Merchant AR High guanine-cytosine content is not an adaptation to high temperature: a comparative analysis amongst prokaryotes Proc R Soc Lond B Biol Sci 2001 268 1466 493 497 10.1098/rspb.2000.1397 PMC1088632 11296861

[B6] Duret L Arndt PF The impact of recombination on nucleotide substitutions in the human genome PLoS Genet 2008 4 5 e1000071 10.1371/journal.pgen.1000071 18464896 PMC2346554

[B7] Meunier J Duret L Recombination drives the evolution of GC-content in the human genome Mol Biol Evol 2004 21 6 984 990 10.1093/molbev/msh070 14963104

[B8] Wang HC Xia XH Hickey D Thermal adaptation of the small subunit ribosomal RNA gene: A comparative study J Mol Evol 2006 63 1 120 126 10.1007/s00239-005-0255-4 16786438

[B9] Varriale A Torelli G Bernardi G Compositional properties and thermal adaptation of 18 S rRNA in vertebrates RNA 2008 14 8 1492 1500 10.1261/rna.957108 18567811 PMC2491464

[B10] Farias ST Bonato MC Preferred amino acids and thermostability Genet Mol Res 2003 2 4 383 393 15011142

[B11] Haney PJ Badger JH Buldak GL Reich CI Woese CR Olsen GJ Thermal adaptation analyzed by comparison of protein sequences from mesophilic and extremely thermophilic Methanococcus species Proc Natl Acad Sci USA 1999 96 7 3578 3583 10.1073/pnas.96.7.3578 10097079 PMC22336

[B12] Kreil DP Ouzounis CA Identification of thermophilic species by the amino acid compositions deduced from their genomes Nucleic Acids Res 2001 29 7 1608 1615 10.1093/nar/29.7.1608 11266564 PMC31282

[B13] Tekaia F Yeramian E Dujon B Amino acid composition of genomes, lifestyles of organisms, and evolutionary trends: a global picture with correspondence analysis Gene 2002 297 1-2 51 60 10.1016/S0378-1119(02)00871-5 12384285

[B14] Zeldovich KB Berezovsky IN Shakhnovich EI Protein and DNA sequence determinants of thermophilic adaptation PLoS Comput Biol 2007 3 1 62 72 10.1371/journal.pcbi.0030005 PMC1769408 17222055

[B15] Glyakina AV Garbuzynskiy SO Lobanov MY Galzitskaya OV Different packing of external residues can explain differences in the thermostability of proteins from thermophilic and mesophilic organisms Bioinformatics 2007 23 17 2231 2238 10.1093/bioinformatics/btm345 17599925

[B16] Cambillau C Claverie JM Structural and genomic correlates of hyperthermostability J Biol Chem 2000 275 42 32383 32386 10.1074/jbc.C000497200 10940293

[B17] Suhre K Claverie JM Genomic correlates of hyperthermostability, an update J Biol Chem 2003 278 19 17198 17202 10.1074/jbc.M301327200 12600994

[B18] Webb CO Ackerly DD Kembel SW Phylocom: software for the analysis of phylogenetic community structure and trait evolution Bioinformatics 2008 24 18 2098 2100 10.1093/bioinformatics/btn358 18678590

[B19] De Vendittis E Castellano I Cotugno R Ruocco MR Raimo G Masullo M Adaptation of model proteins from cold to hot environments involves continuous and small adjustments of average parameters related to amino acid composition J Theor Biol 2008 250 1 156 171 10.1016/j.jtbi.2007.09.006 17950361

[B20] Saelensminde G Halskau O Helland R Willassen NP Jonassen I Structure-dependent relationships between growth temperature of prokaryotes and the amino acid frequency in their proteins Extremophiles 2007 11 4 585 596 10.1007/s00792-007-0072-3 17429573

[B21] Cambillau C Claverie JM Structural and genomic correlates of hyperthermostability J Biol Chem 2000 275 42 32383 32386 10.1074/jbc.C000497200 10940293

[B22] Shedlock AM Botka CW Zhao S Shetty J Zhang T Liu JS Deschavanne PJ Edwards SV Phylogenomics of nonavian reptiles and the structure of the ancestral amniote genome Proc Natl Acad Sci USA 2007 104 8 2767 2772 10.1073/pnas.0606204104 17307883 PMC1815256

[B23] Sabbia V Piovani R Naya H Rodriguez-Maseda H Romero H Musto H Trends of amino acid usage in the proteins from the human genome J Biomol Struct Dyn 2007 25 1 55 59 17676938 10.1080/07391102.2007.10507155

[B24] Bicego KC Barros RC Branco LG Physiology of temperature regulation: comparative aspects Comp Biochem Physiol A Mol Integr Physiol 2007 147 3 616 639 10.1016/j.cbpa.2006.06.032 16950637

[B25] Berezovsky IN Shakhnovich EI Physics and evolution of thermophilic adaptation Proc Natl Acad Sci USA 2005 102 36 12742 12747 10.1073/pnas.0503890102 16120678 PMC1189736

[B26] Mizuguchi K Sele M Cubellis MV Environment specific substitution tables for thermophilic proteins BMC Bioinformatics 2007 8 Suppl 1 S15 10.1186/1471-2105-8-S1-S15 17430559 PMC1885844

[B27] Ciccarelli FD Doerks T von Mering C Creevey CJ Snel B Bork P Toward automatic reconstruction of a highly resolved tree of life Science 2006 311 5765 1283 1287 10.1126/science.1123061 16513982

[B28] Edgar RC MUSCLE: multiple sequence alignment with high accuracy and high throughput Nucleic Acids Res 2004 32 5 1792 1797 10.1093/nar/gkh340 15034147 PMC390337

[B29] Talavera G Castresana J Improvement of phylogenies after removing divergent and ambiguously aligned blocks from protein sequence alignments Syst Biol 2007 56 4 564 577 10.1080/10635150701472164 17654362

[B30] Guindon S Gascuel O A simple, fast, and accurate algorithm to estimate large phylogenies by maximum likelihood Syst Biol 2003 52 5 696 704 10.1080/10635150390235520 14530136

