# Supplementary material for: Amino acid composition in endothermic vertebrates is biased in the same direction as in thermophilic prokaryotes
Source: BMC Evol Biol. 2010 Aug 31;10:263. doi: 10.1186/1471-2148-10-263 (PMC2939578; doi:10.1186/1471-2148-10-263)
Supplement: Additional file 1 — Supplemental Figures and Tables. Additional results that support the conclusions of the main text, presented in the form of figures and tables. [file 1471-2148-10-263-S1.PDF]

Supplemental Material

Amino acid composition in endothermic vertebrates is biased in the same direction as in thermophilic prokaryotes

Guang-Zhong Wang & Martin J. Lercher

Supplemental Figures 2

Supplemental Tables 5

Supplemental Figures

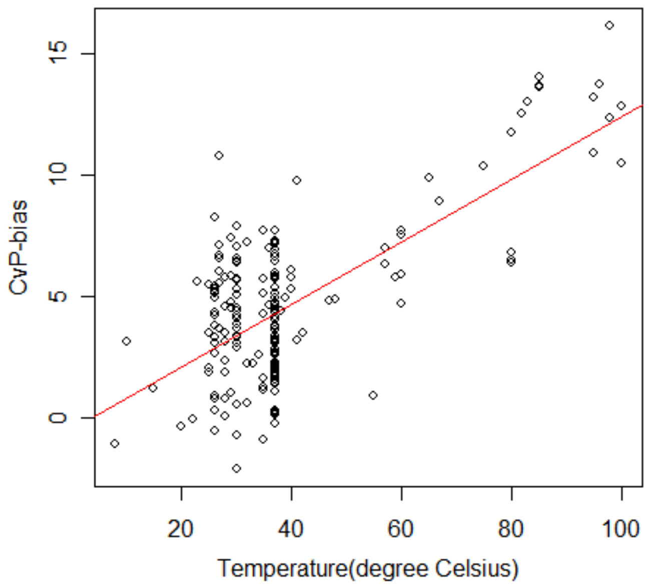

Figure S1. Correlation between an alternative measure of amino acid bias related to protein stability, *CvP-bias*= D +E+R+K-N-Q-T-S, and optimal growth temperature in 204 prokaryotes (Pearson's R=0. 69, $p< 10^{-15}$; Spearman's ρ =0. 31, $p=5\times10^{-6}$).

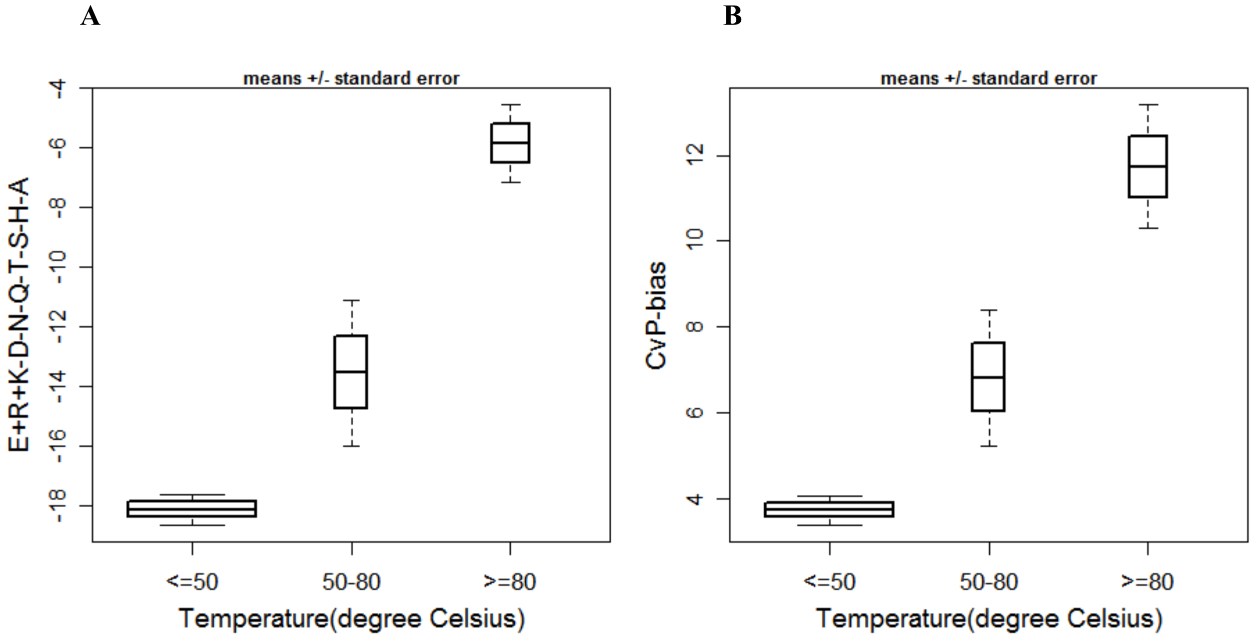

Figure S2. Differences between measures of amino acid bias related to protein stability and optimal growth temperature in hyperthermophiles (optimal growth temperature OGT≥80°C), thermophiles (OGT=50-80 °C), and mesophiles (OGT≤50°C), using either *ERK*=E+R+K-D-N-Q-T-S-H-A (**A**) or *CvP-bias*=D+E+R+K-N-Q-T-S (**B**) as measures of amino acid bias. All pairwise comparisons show highly significant differences (Wilcoxon rank sum tests for *ERK* (*CvP-bias*): $p=2\times10^{-5}$ (0.00023) between hyperthermophiles and thermophiles, p=0.00059 (0.00024) between thermophiles and mesophiles, and $p=4\times10^{-11}$ (2×10^{-10}) between hyperthermophiles and mesophiles). Central lines are means, boxes give standard error, whiskers 2x standard error.

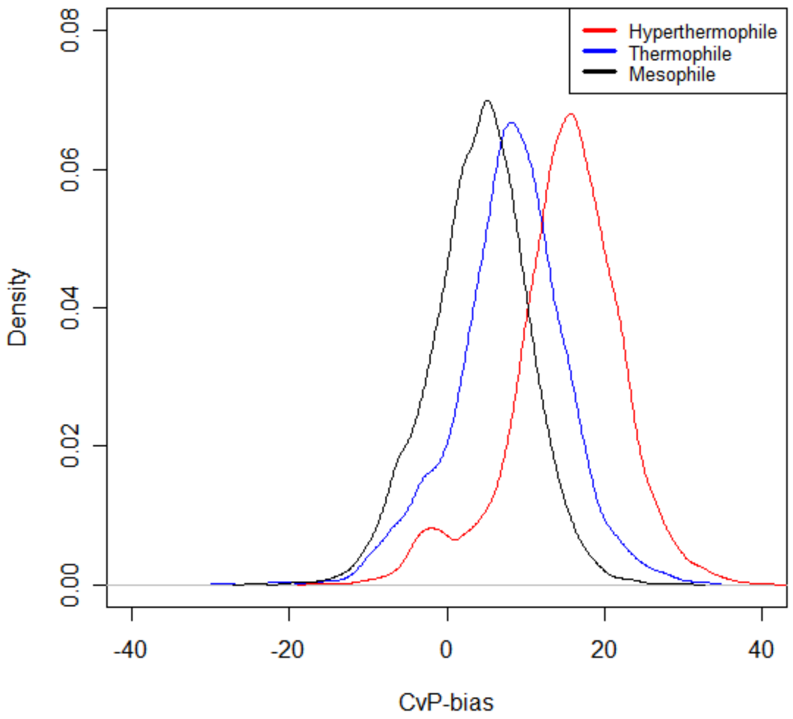

Figure S3. Distribution of *CvP-bias* across orthologous proteins for 5 species each of hyperthermophile (optimal growth temperature ≥80˚ Celsius), thermophile (50-80˚), and mesophile (≤50˚) prokaryotes.

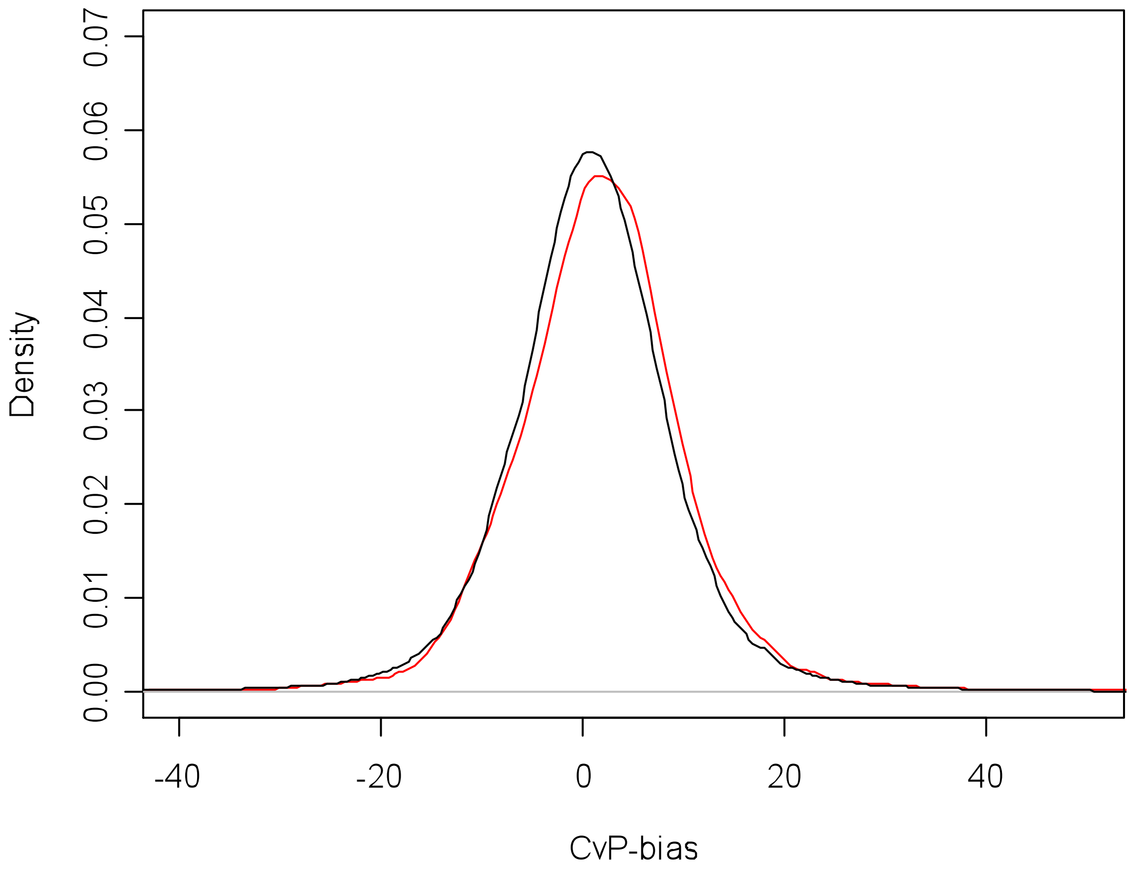

Figure S4. Distribution of an alternative measure of amino acid bias, CvP-bias, across proteins for warm-blooded vertebrates (red line: mammals, birds) and cold-blooded vertebrates (black line: Reptilia, Amphibia, fish). *CvP-bias* is significantly increased in warm-blooded relative to cold-blooded animals (Wilcoxon rank sum test: $p<10^{-15}$).

Supplemental Tables

Table S1. Summary of the 15 prokaryotic genomes used for the calculation of co-orthologs in this study (see Methods for details). OGT=optimal growth temperature.

Life style	Species	Kingdom	OGT(°C)	Number of orthologs
Hyper-thermophiles	Aquifex aeolicus	Bacteria	90	5334
	Methanopyrus kandleri	Archaea	98	
	Methanococcus jannaschii	Archaea	85	
	Pyrococcus abyssi	Archaea	97	
	Pyrococcus furiosus	Archaea	98	
Thermophiles	Thermoplasma volcanium	Archaea	60	5366
	Thermoplasma acidophilum	Archaea	58	
	Methanobacterium thermoautotrophicum	Archaea	65	
	Carboxydothermus hydrogenoformans Z-2901	Bacteria	68	
	Chloroflexus aurantiacus J_10_fl	Bacteria	50-60	
Mesophiles	Haemophilus influenzae	Bacteria	37	4593
	Staphylococcus aureus Mu50	Bacteria	37	
	Corynebacterium glutamicum ATCC 13032 Bielefeld	Bacteria	30	
	Mycobacterium tuberculosis H37Rv	Bacteria	37	
	Salmonella typhi	Bacteria	37	

Table S2. *P*-value of two measures of amino acid usage bias, *ERK* and CvP-*bias,* across all codons of orthologous proteins for warm-blooded and cold-blooded animals. *ERK* and CvP-*bias* are significantly higher in warm-blooded genomes compared to *Anolis carolinensis* (1-sided Wilcoxon rank sum tests).

Class	Species	*p* (*ERK*)	*p* (*CvP-bias*)
Mammalia	Mus musculus	0.0014	0.00045
Mammalia	Rattus norvegicus	0.0093	0.012
Mammalia	Human	$4.9 X10^{-16}$	$1.3 X10^{-11}$
Mammalia	Bos taurus	$2.2X10^{-16}$	$2.2X10^{-16}$
Birds	Gallus gallus	$2.2X10^{-16}$	$2.2X10^{-16}$
Amphibia	Xenopus laevis	0.081	1
Amphibia	Xenopus tropicalis	0.10	1
Fish	Danio rerio	1	1
Fish	Tetraodon nigroviridis	1	1
Fish	Takifugu rubripes	1	1

Table S3. Comparison of orthologous protein fragments from *Gallus gallus* and three reptilian species *p*-values are from Wilcoxon paired rank tests, comparing mean *ERK* or *CVP-bias* of chicken and the respective reptile.

Reptilia species	Number of protein fragments	Reptile *ERK*	Chicken *ERK*	*p*	Reptile *CvP-bias*	Chicken *CvP-bias*	*p*
Alligator	508	-10.34	-7.41	$4 x10^{-06}$	7.76	11.53	$2 x10^{-16}$
Lizard	138	-13.53	-11.07	0.00027	4.24	7.64	$2 x10^{-07}$
Turtle	429	-11.64	-10.85	0.011	4.91	7.21	$7 x10^{-12}$

Table S4. List of species used in the analysis controlling for phylogenetic non-independence

Species	OGT
Photobacterium profundum	15
Agrobacterium tumefaciens WashU	26
Bradyrhizobium japonicum	26
Fusobacterium nucleatum subsp. nucleatum	26
Mesorhizobium loti	26
Mycoplasma mobile 163K	26
Nitrosomonas europaea	26
Pseudomonas syringae pv. tomato	26
Sinorhizobium meliloti	26
Synechococcus elongatus	26
Xanthomonas campestris pv. campestris	26
Xylella fastidiosa	26
Streptomyces avermitilis	27
Streptomyces coelicolor	27
Oceanobacillus iheyensis	28
Pseudomonas putida KT2440	28
Ralstonia solanacearum	28
Yersinia pestis KIM	28
Borrelia burgdorferi	29
Photorhabdus luminescens subsp. laumondii	29
Bacillus anthracis str. Ames	30
Bacillus halodurans	30
Bdellovibrio bacteriovorus	30
Chromobacterium violaceum	30
Deinococcus radiodurans	30
Desulfovibrio vulgaris subsp. vulgaris str. Hildenborough	30
Lactobacillus plantarum	30
Lactococcus lactis subsp. lactis	30
Leptospira interrogans serovar Copenhageni	30
Rhodopseudomonas palustris	30
Shewanella oneidensis	30
Xanthomonas axonopodis pv. citri	30
Haemophilus ducreyi	32
Corynebacterium glutamicum ATCC 13032	33
Methanococcus maripaludis	35
Rickettsia conorii	35
Rickettsia prowazekii	35
Bordetella bronchiseptica	36
Methanosarcina mazei	36
Bacillus subtilis	37
Bacteroides thetaiotaomicron	37
Bifidobacterium longum	37
Bordetella parapertussis	37
Bordetella pertussis	37
Brucella melitensis	37

Chlamydia muridarum	37
Chlamydia trachomatis	37
Chlamydophila pneumoniae AR39	37
Clostridium acetobutylicum	37
Clostridium perfringens	37
Clostridium tetani	37
Corynebacterium diphtheriae	37
Corynebacterium efficiens	37
Enterococcus faecalis	37
Escherichia coli	37
Haemophilus influenzae	37
Halobacterium sp. NRC-1	37
Helicobacter hepaticus	37
Helicobacter pylori	37
Lactobacillus johnsonii	37
Listeria innocua	37
Listeria monocytogenes	37
Mycobacterium bovis	37
Mycobacterium leprae	37
Mycobacterium tuberculosis CDC1551	37
Mycoplasma gallisepticum	37
Mycoplasma genitalium	37
Mycoplasma penetrans	37
Mycoplasma pneumoniae	37
Mycoplasma pulmonis	37
Pasteurella multocida	37
Porphyromonas gingivalis	37
Salmonella enterica subsp. enterica serovar Typhi Ty2	37
Salmonella typhi	37
Shigella flexneri	37
Shigella flexneri 2a str. 2457T	37
Staphylococcus epidermidis	37
Streptococcus agalactiae serogroup III	37
Streptococcus mutans	37
Streptococcus pneumoniae R6	37
Streptococcus pyogenes	37
Treponema denticola	37
Treponema pallidum	37
Vibrio cholerae	37
Vibrio parahaemolyticus	37
Vibrio vulnificus	37
Wolinella succinogenes	37
Mycobacterium avium subsp. paratuberculosis	39
Campylobacter jejuni	40
Methanosarcina acetivorans	40
Pseudomonas aeruginosa	40
Chlorobaculum tepidum	48

Thermoplasma acidophilum	59
Thermoplasma volcanium	60
Thermoanaerobacter tengcongensis	75
Sulfolobus solfataricus	80
Sulfolobus tokodaii	80
Thermotoga maritima	80
Nanoarchaeum equitans	82
Archaeoglobus fulgidus	83
Aquifex aeolicus	85
Methanocaldococcus jannaschii	85
Thermus thermophilus HB27	85
Aeropyrum pernix	95
Pyrococcus abyssi	96
Methanopyrus kandleri	98
Pyrococcus horikoshii	98
Pyrobaculum aerophilum	100
Pyrococcus furiosus	100
